# A Calculus of Purpose

**DOI:** 10.1371/journal.pbio.0020164

**Published:** 2004-06-15

**Authors:** Arthur D Lander

## Abstract

Biological systems are so complex that we must ask: "What purpose does all this complexity serve?" Lander argues that computational biology may help provide answers

Why is the sky blue? Any scientist will answer this question with a statement of mechanism: Atmospheric gas scatters some wavelengths of light more than others. To answer with a statement of purpose—e.g., to say the sky is blue in order to make people happy—would not cross the scientific mind. Yet in biology we often pose “why” questions in which it is purpose, not mechanism, that interests us. The question “Why does the eye have a lens?” most often calls for the answer that the lens is there to focus light rays, and only rarely for the answer that the lens is there because lens cells are induced by the retina from overlying ectoderm.

It is a legacy of evolution that teleology—the tendency to explain natural phenomena in terms of purposes—is deeply ingrained in biology, and not in other fields (Ayala 1999). Natural selection has so molded biological entities that nearly everything one looks at, from molecules to cells, from organ systems to ecosystems, has (at one time at least) been retained because it carries out a function that enhances fitness. It is natural to equate such functions with purposes. Even if we can't actually know why something evolved, we care about the useful things it does that could account for its evolution.

As a group, molecular biologists shy away from teleological matters, perhaps because early attitudes in molecular biology were shaped by physicists and chemists. Even geneticists rigorously define function not in terms of the useful things a gene does, but by what happens when the gene is altered. Molecular biology and molecular genetics might continue to dodge teleological issues were it not for their fields' remarkable recent successes. Mechanistic information about how a multitude of genes and gene products act and interact is now being gathered so rapidly that our inability to synthesize such information into a coherent whole is becoming more and more frustrating. Gene regulation, intracellular signaling pathways, metabolic networks, developmental programs—the current information deluge is revealing these systems to be so complex that molecular biologists are forced to wrestle with an overtly teleological question: What purpose does all this complexity serve?

In response to this situation, two strains have emerged in molecular biology, both of which are sometimes lumped under the heading “systems biology.” One strain, bioinformatics, champions the gathering of even larger amounts of new data, both descriptive and mechanistic, followed by computerbased data “mining” to identify correlations from which insightful hypotheses are likely to emerge. The other strain, computational biology, begins with the complex interactions we already know about, and uses computer-aided mathematics to explore the consequences of those interactions. Of course, bioinformatics and computational biology are not entirely separable entities; they represent ends of a spectrum, differing in the degree of emphasis placed on large versus small data sets, and statistical versus deterministic analyses.

Computational biology, in the sense used above, arouses some skepticism among scientists. To some, it recalls the “mathematical biology” that, starting from its heyday in the 1960s, provided some interesting insights, but also succeeded in elevating the term “modeling” to near-pejorative status among many biologists. For the most part, mathematical biologists sought to fit biological data to relatively simple mathematical models, with the hope that fundamental laws might be recognized (Fox Keller 2002). This strategy works well in physics and chemistry, but in biology it is stymied by two problems. First, biological data are usually incomplete and extremely imprecise. As new measurements are made, today's models rapidly join tomorrow's trash heaps. Second, because biological phenomena are generated by large, complex networks of elements, there is little reason to expect to discern fundamental laws in them. To do so would be like expecting to discern the fundamental laws of electromagnetism in the output of a personal computer.

Nowadays, many computational biologists avoid modeling-as-data-fitting, opting instead to create models in which networks are specified in terms of elements and interactions (the network “topology”), but the numerical values that quantify those interactions (the parameters) are deliberately varied over wide ranges. As a result, the study of such networks focuses not on the exact values of outputs, but rather on qualitative behavior, e.g., whether the network acts as a “switch,” “filter,” “oscillator,” “dynamic range adjuster,” “producer of stripes,” etc. By investigating how such behaviors change for different parameter sets— an exercise referred to as “exploring the parameter space”—one starts to assemble a comprehensive picture of all the kinds of behaviors a network can produce. If one such behavior seems useful (to the organism), it becomes a candidate for explaining why the network itself was selected, i.e., it is seen as a potential purpose for the network. If experiments subsequently support assignments of actual parameter values to the range of parameter space that produces such behavior, then the potential purpose becomes a likely one.

For very simple networks (e.g., linear pathways with no delays or feedback and with constant inputs), possible global behaviors are usually limited, and computation rarely reveals more than one could have gleaned through intuition alone. In contrast, when networks become even slightly complex, intuition often fails, sometimes spectacularly so, and computation becomes essential.

For example, intuitive thinking about MAP kinase pathways led to the long-held view that the obligatory cascade of three sequential kinases serves to provide signal amplification. In contrast, computational studies have suggested that the purpose of such a network is to achieve extreme positive cooperativity, so that the pathway behaves in a switch-like, rather than a graded, fashion (Huang and Ferrell 1996). Another example comes from the study of morphogen gradient formation in animal development. Whereas intuitive interpretations of experiments led to the conclusion that simple diffusion is not adequate to transport most morphogens, computational analysis of the same experimental data yields the opposite conclusion (Lander et al. 2002).

As the power of computation to identify possible functions of complex biological networks is increasingly recognized, purely (or largely) computational studies are becoming more common in biological journals. This raises an interesting question for the biology community: In a field in which scientific contributions have long been judged in terms of the amount of new experimental data they contain, how does one judge work that is primarily focused on interpreting (albeit with great effort and sophistication) the experimental data of others? At the simplest level, this question poses a conundrum for journal editors. At a deeper level, it calls attention to the biology community's difficulty in defining what, exactly, constitutes “insight” (Fox Keller 2002).

In yesterday's mathematical biology, a model's utility could always be equated with its ability to generate testable predictions about new experimental outcomes. This approach works fine when one's ambition is to build models that faithfully mimic particular biological phenomena. But when the goal is to identify all possible classes of biological phenomena that could arise from a given network topology, the connection to experimental verification becomes blurred. This does not mean that computational studies of biological networks are disconnected from experimental reality, but rather that they tend, nowadays, to address questions of a higher level than simply whether a particular model fits particular data.

The problem this creates for those of us who read computational biology papers is knowing how to judge when a study has made a contribution that is deep, comprehensive, or enduring enough to be worth our attention. We can observe the field trying to sort out this issue in the recent literature. A good example can be found in an article by Nicholas Ingolia in this issue of *PLoS Biology* (Ingolia 2004), and an earlier study from Garrett Odell's group, upon which Ingolia draws heavily (von Dassow et al. 2000).

Both articles deal with a classical problem in developmental biology, namely, how repeating patterns (such as stripes and segments) are laid down. In the early fruit fly embryo, it is known that a network involving cell-to-cell signaling via the Wingless (Wg) and Hedgehog (Hh) pathways specifies the formation and maintenance of alternating stripes of gene expression and cell identity. This network is clearly complex, in that Wg and Hh signals affect not only downstream genes, but also the expression and/or activity of the components of each other's signaling machinery.

Von Dassow et al. (2000) calculated the behaviors of various embodiments of this network over a wide range of parameter values and starting conditions. This was done by expressing the network in terms of coupled differential equations, picking parameters at random from within prespecified ranges, solving the equation set numerically, then picking another random set of parameters and obtaining a new numerical solution, and so forth, until 240,000 cases were tried. The solutions were then sorted into groups based on the predicted output—in this case, spatial patterns of gene expression.

When they used a network topology based only upon molecular and generegulatory interactions that were firmly known to take place in the embryo, they were unable to produce the necessary output (stable stripes), but upon inclusion of two molecular events that were strongly suspected of taking place in the embryo, they produced the desired pattern easily. In fact, they produced it much more easily than expected. It appeared that a remarkably large fraction of random parameter values produced the very same stable stripes. This implied that the output of the network is extraordinarily robust, where robustness is meant in the engineering sense of the word, namely, a relative insensitivity of output to variations in parameter values.

Because real organisms face changing parameter values constantly—whether as a result of unstable environmental conditions, or mutations leading to the inactivation of a single allele of a gene—robustness is an extremely valuable feature of biological networks, so much so that some have elevated it to a sort of sine qua non (Morohashi et al. 2002). Indeed, the major message of the von Dassow article was that the authors had uncovered a “robust developmental module,” which could ensure the formation of an appropriate pattern even across distantly related insect species whose earliest steps of embryogenesis are quite different from one another (von Dassow et al. 2000).

There is little doubt that von Dassow's computational study extracted an extremely valuable insight from what might otherwise seem like a messy and ill-specified system. But Ingolia now argues that something further is needed. He proposes that it is not enough to show that a network performs in a certain way; one should also find out why it does so.

Ingolia throws down the gauntlet with a simple hypothesis about why the von Dassow network is so robust. He argues that it can be ascribed entirely to the ability of two positive feedback loops within the system to make the network bistable. Bistability is the tendency for a system's output to be drawn toward either one or the other of two stable states. For example, in excitable cells such as neurons, depolarization elicits sodium entry, which in turn elicits depolarization—a positive feedback loop. As a result, large depolarizations drive neurons to fully discharge their membrane potential, whereas small depolarizations decay back to a resting state. Thus, the neuron tends strongly toward one or the other of these two states. The stability of each state brings with it a sort of intrinsic robustness— i.e., once a cell is in one state, it takes a fairly large disturbance to move it into the other. This is the same principle that makes electronic equipment based on digital (i.e., binary) signals so much more resistant to noise than equipment based on analog circuitry.

Ingolia not only argues that robustness in the von Dassow model arises because positive feedback leads to network bistability, he further claims that such network bistability is a consequence of bistability at the single cell level. He strongly supports these claims through computational explorations of parameter space that are similar to those done by von Dassow et al., but which also use strippeddown network topologies (to focus on individual cell behaviors), test specifically for bistability, correlate results with the patterns formed, and ultimately generate a set of mathematical rules that strongly predict those cases that succeed or fail at producing an appropriate pattern.

At first glance, such a contribution might seem no more than a footnote to von Dassow's paper, but a closer look shows that this is not the case. Without mechanistic information about why the von Dassow network does what it does, it is difficult to relate it to other work, or to modify it to accommodate new information or new demands. Ingolia demonstrates this by deftly improving on the network topology. He inserts some new data from the literature about the product of an additional gene, *sloppy-paired*, in Hh signaling, removes some of the more tenuous connections, and promptly recovers a biologically essential behavior that the original von Dassow network lacked: the ability to maintain a fixed pattern of gene expression even in the face of cell division and growth.

Taken as a pair, the von Dassow and Ingolia papers illustrate the value of complementary approaches in the analysis of complex biological systems. Whereas one emphasizes simulation (as embodied in the numerical solution of differential equations), the other emphasizes analysis (the mathematical analysis of the behavior of a set of equations). Whereas one emphasizes exploration (exploring a parameter space), the other emphasizes the testing of hypotheses (about the origins of robustness). The same themes can be seen in sets of papers on other topics. For example, in their analysis of bacterial chemotaxis, Leibler and colleagues (Barkai and Leibler 1997) found a particular model to be extremely robust in the production of an important behavior (exact signal adaptation), and subsequently showed that bacteria do indeed exhibit such robust adaptation (Alon et al. 1999). Although Leibler and colleagues took significant steps toward identifying and explaining how such robustness came about, it took a subsequent group (Yi et al. 2000) to show that robustness emerged as a consequence of a simple engineering design principle known as “integral feedback control.” That group also showed, through mathematical analysis, that integral feedback control is the only feedback strategy capable of achieving the requisite degree of robustness.

From these and many other examples in the literature, one can begin to discern several of the elements that, when present together, elevate investigations in computational biology to a level at which ordinary biologists take serious notice. Such elements include network topologies anchored in experimental data, fine-grained explorations of large parameter spaces, identification of “useful” network behaviors, and hypothesisdriven analyses of the mathematical or statistical bases for such behaviors. These elements can be seen as the foundations of a new calculus of purpose, enabling biologists to take on the much-neglected teleological side of molecular biology. “What purpose does all this complexity serve?” may soon go from a question few biologists dare to pose, to one on everyone's lips.
